# Prevalence and patterns of birth defects among newborns in southwestern Ethiopia: a retrospective study

**DOI:** 10.11604/pamj.2021.40.248.25286

**Published:** 2021-12-21

**Authors:** Soressa Abebe Geneti, Girmai Gebru Dimsu, Demisew Amenu Sori, Lemessa Dube Amente, Zeleke Mekonnen Kurmane

**Affiliations:** 1 Department of Anatomy, School of Medicine, College of Health Sciences, Addis Ababa University, Addis Ababa, Ethiopia,; 2 Department of Obstetrics and Gynecology, School of Medicine, Institute of Health, Jimma University, Jimma, Ethiopia,; 3 Department of Epidemiology, School of Public Health, Institute of Health, Jimma University, Jimma, Ethiopia,; 4 School of Medical Laboratory Sciences, Institute of Health, Jimma University, Jimma, Ethiopia

**Keywords:** Birth defect, prevalence, frequency, Ethiopia

## Abstract

**Introduction:**

prenatal development could be considered normal or abnormal. Abnormal development occurs because of interference of normal development from genetic disorders, environmental factors, and multifactorial inheritances during the critical period of embryogenesis. The present study was aimed at evaluating the prevalence and patterns of birth defects among newborns in southwestern Ethiopia.

**Methods:**

institutions-based cross-sectional study design was conducted in six purposively selected hospitals in southwestern Ethiopia based on their caseload. The study included data's from 2011 to 2015, during which 45,951 deliveries were recorded. All records of births in the selected hospitals were screened from medical records to identify the presence and types of birth defects.

**Results:**

out of twenty-one different birth defects identified, nearly half of them belong to anencephaly and hydrocephalus. Five types of birth defects, namely: anencephaly (25.0%), hydrocephalus (24.6%), spina bifida (13.1%), meningomyelocele (7.1%), and umbilical hernia (4.8%) accounted for about three-fourths (75%) of all recorded birth defects. The prevalence rate of birth defects at birth was 55 per 10,000 births.

**Conclusion:**

in the present study, the neural tube defects were identified to be the most prevalent. Nearly equal proportions of birth defects occurred among male and female newborns. The majority of the mothers who gave birth to newborns with birth defects were younger than 35 years. The high prevalence of birth defects revealed in this study call for the need to implement urgent prevention strategies including but not limited to the provision of sustained family planning, youth education and antenatal care services, and strict observation of rational medication use during pregnancy to curb the possible occurrences of the birth defect.

## Introduction

Prenatal development can be considered either normal or an abnormal development. Abnormal development occurs because of the interference of normal development from genetic disorders, environmental factors, or the combination of both of these, genetic and environmental, factors during the critical period of embryogenesis. These factors may lead to abnormal cytogenesis, histogenesis, and morphogenesis with which the neonate are born with defect [1,2].

Congenital anomaly (CA), commonly called birth defect (BD), is a structural and functional abnormality that presents at birth. BD can be described in terms of four clinically significant types based on the causes, timing, and extent of the developmental interference during prenatal life by teratologic agents [3]. These types are malformation, disruption, deformation, and dysplasia [4,5]. The World Health Organization (WHO) estimates that approximately 260,000 deaths (7% of all neonatal deaths) globally were caused by BDs in 2004 [6]. It is estimated that the prevalence rates of BDs are 4.7% in the developed countries, 5.6% in the middle-income countries, and 6.4% in the low-income countries [7].

The prevalence of BD varies among different ethnic groups. Extensive literature is available on the incidence of BDs in first-world countries and several third-world countries [8]. However, in sub-Saharan African countries including Ethiopia, limited information is available on the prevalence of BDs. Moreover, the limited number of studies suffer from several limitations such as retrospective study design, small sample size and study objectives limited to identifying the frequency of a single BD and/or a few specific defcts [1].

The significant variability in the documented frequency and types of BDs in different parts of Ethiopia and other countries might be due to the differences in genetic background and geographic location, nutritional and socioeconomic statuses [9]. BDs begin to emerge as one of the significant childhood health problems and a cause of infant death, especially in developing countries [9,10]. In most cases, infants with the defects do not survive, and more than 70% die in the first month of their live [11-15].

The frequency of BDs in developing countries, including Ethiopia, is underestimated because of a lack of diagnostic capabilities, complete medical records, and health statistics. Notwithstanding the limitations associated with utilizing diagnostic data that are solely based on apparent illnesses, the available recorded diagnoses in vital health statistics rely on such data rather than on preexisting BDs present at birth. This fact increases infant mortality and morbidity, leading to an underestimate of the true magnitude of the problem [1,16,17]. BDs are the fifth leading cause of years of potential life loss and significant causes of infant deaths throughout the world [18]. Despite of their clinical importance, few studies have been conducted on the prevalence of BDs and predisposing risk factors [11,19-21]. Consequently, the frequency, types, and patterns of BDs need to be evaluated. Hence, the present study was aimed at determining the prevalence and patterns of birth defects at birth in southwestern Ethiopia.

## Methods

**Study setting:** the study was conducted in six selected hospitals in three zones in southwestern Ethiopia, namely, Jimma Medical Centre, Shanan Gibe hospital, Limmu Genet hospital, and Agaro hospital in Jimma zone; Mattu Karl hospital in Ilu Abba Bori zone and Nekemte hospital in East Wollega zone. The three zones are among the seventeen zones in the west Oromia national regional state, the largest regional state in Ethiopia. The study was conducted retrospectively from 2011 to 2015.

**Study design and sampling:** an institutional-based cross-sectional study design was conducted in six purposively selected hospitals with high caseloads among the eight available hospitals. The sampling frame included all deliveries attended in the selected hospitals from 2011 to 2015, during which a total of 45,951 deliveries were attended in the hospitals. The sampling population involved all deliveries with BDs that included both live and stillbirths. A total of 44,991 records was the minimum sample size estimated according to Dean *et al*. [22] with the assumptions that: proportion (p) of BD in Ethiopia to be 20.0 per 1000 birth [23], confidence interval 95%, degree of precision (d), 0.013%. The absolute precision was reduced from the conventional 5% as the BDs are very rare events. To get the required sample, five years of medical records were reviewed, which was a bit greater than the estimated sample size.

**Data collection:** based on the routine and national guidelines, all newborn babies were routinely screened for BD immediately after birth and before they were discharged from the hospitals. Physicians performed general physical examination of the newborns and screened for the presence of BD before discharge. Then, the midwife on duty carried out the discharge as per the order and recorded the information on the nationally developed registration logbooks. Consequently, the records of all newborn babies from 2011 to 2015 were reviewed. Data were extracted from the logbooks and transcribed into a standard checklist that was adapted from data collection instruments used in similar previous studies. Accordingly, we have registered the card numbers of newborns with BDs from the logbooks and reviewed the records against the developed standard checklist. Trained midwives, nurses, and general practitioners collected the necessary data needed for the study. We have retrieved variables like the type of BD, socio-demographic characteristics (age, educational status, income, residence, religion of the mother), and obstetric history (parity, gravida, history of abortion and stillbirth, gestational age, and birth weight) to characterize the newborn babies.

**Measurements:** the types and prevalence of BDs were the outcome or dependent variables of the present study. The association of the status of an infant at birth, birth weight, and gestational age with the types of BDs was also the secondary outcome of this study. The types of BDs were classified according to ICD-10 (International Classification of Diseases - 10). Total prevalence was calculated by dividing the numerator (registered cases of BDs) by the relevant denominator (total live and stillbirths) for the same period at the same place multiplied by 1000/10,000.

**Statistical analysis:** the collected data were checked regularly for their completeness and correctness, then entered into EpiData Manager Version 2.0.8.56 computer software. The data were then exported to SPSS software program version 22.0 for analysis. Descriptive statistics like frequency was used to describe the study participants. A newborn with more than one anomaly was counted once only based on the primary diagnosis. A P-value of less than 0.05 was considered as statistically significant using the Fisher exact test.

**Ethical approval:** ethical approval was obtained from the Institutional Review Board of the College of Health Sciences (IRB-CHS), Addis Ababa University, and Jimma University Institutional Review Board to conduct the research. We submitted the ethical and supportive letters to medical directors of the respective study hospitals and obtained permission for data collection. The data collected from the medical records were kept confidential.

## Results

The overall prevalence of BDs was 55 per 10,000 births during the study period in the study areas, during which a total of 45,951 deliveries were recorded, and out of these, 253 births were with BDs. Among the 253 births with BDs, 252 were with sex determined (51.38% males and 48.22% females) while, one (0.4%) with ambiguous genitalia at birth. The majority (92%) of the mothers were younger than 35 years. About half of the defects (49.6%) occurred among mothers whose ages were between 25 - 35 years. About 63.1% of fetuses born with BDs were stillbirths. The majority, 92.5%, and 93.3%, of the mothers, respectively had no previous history of abortion and stillbirth (Table 1).

**Table 1:** socio-demographic and obstetrics characteristics of mothers and infants born with birth defects from 2011 to 2015 in southwestern Ethiopia

Characteristics		Frequency (%)	Percentage
The religion of the mothers	Muslim	115	45.6
	Orthodox	33	13.1
	Protestant	26	10.3
	Not mentioned	78	31.1
Age of the mothers	15-24	105	41.7
	25-35	125	49.6
	36-43	12	4.8
	Missing	10	4.0
Onset of labor	Spontaneous	202	80.2
	Induced	50	19.9
Mode of delivery	Vaginal	213	84.5
	Cesarean section	39	15.5
Sex of the newborn	Male	130	51.4
	Female	122	48.2
	Ambiguous genitalia	1	0.4
Status at birth	Alive birth	93	36.9
	Stillbirth	159	63.1
History of abortion	Yes	19	7.5
	No	233	92.5
History of stillbirth	Yes	17	6.7
	No	235	93.3
Gestational age	Term	139	55.2
	Preterm	99	39.3
	Postterm	3	1.2
	Missing	11	4.4
Birth order	1-4	116	46.0
	5-9	41	16.3
	Missing	95	37.7

Out of twenty-one different types of BDs observed in this study, neural tube defect (NTD) was the most prevalent with the frequency of 73.5%, followed by gastrointestinal defects (13.4%) and musculoskeletal (11.1%). Genitourinary defects were the least prevalent, with a frequency of 2% (Table 2). Of the NTDs recorded, anencephaly (33.9%) and hydrocephalus (33.3%) were the most frequent, followed by spina bifida (17.7%). In contrast, microcephaly and craniorachischisis (each 1.6%) were the least frequent. Among the gastrointestinal defects, congenital umbilical hernia (35.3%) was the most prevalent, while omphalocele (2.9%) was the least one. Others were imperforate anus, gastroschisis, duodenal atresia, and congenital inguinal hernia, accounting for 26.5%, 17.7%, 8.8%, and 8.8%, respectively.

**Table 2:** frequency of birth defects by sex among deliveries conducted from 2011 to 2015 in southwestern Ethiopia

Birth defects	Male ( n = 130)		Female (n = 122)		Total ( n= 253)	
	Frequency	%	Frequency	%	Frequency	%
Neural tube defects	89	67.9	97	79.5	186	73.5
Gastrointestinal defects	20	15.3	14	11.5	34	13.4
Musculoskeletal defects	17	13.0	11	9.0	28	11.1
Genitourinary defects	4	3.1	0	0	4 + 1* = 5	2.0
Total	130	100	122	100	253	100

Among musculoskeletal defects, clubfoot was the commonest (36%) while, cleft lip, cleft palate, both cleft lip and palate, and chest deformity were observed in 24%, 20%, 16%, and 4%, respectively. Genitourinary defects were the least frequent, constituting only 2% of the total BDs identified in this study. Among genitourinary defects, the proportion of hypospadias, meatal stenosis, ambiguous genitalia were 50%, 37.5%, and 12.5%, respectively. The result showed that hypospadias was the most frequent, and ambiguous genitalia was the least frequent. Of the 21 BDs identified in the present study, five types of BDs, namely: anencephaly (25.0%), hydrocephalus (24.6%), spina bifida (13.1%), meningomyelocele (7.1%), and umbilical hernia (4.8%), accounted for about three-fourth (75%) of all recorded BDs (Table 3). The association between the common types of defects and characteristics of the mothers and children was computed using Fisher´s Exact Test. Only the status of the fetus at birth (P-value =0001), birth weight (P-value =0.0001), and gestational age (P-value =0.0001) were found to be associated with types of BDs.

**Table 3:** types and frequency of birth defects recorded among deliveries from 2011 to 2015 in southwestern Ethiopia

Types of Birth defects	Frequency (%)	Percentage
Anencephaly	63	25.0
Hydrocephalus	62	24.6
Spina bifida	33	13.1
Meningomyelocele	18	7.1
Umbilical hernia	12	4.8
Imperforate anus	9	3.6
Clubfoot-bilateral	9	3.6
Cleft lip	6	2.4
Gastroschisis	6	2.4
Both cleft lip and palate	5	2.0
Hypospadias	4	1.6
Cleft palate	4	1.6
Encephalocele	4	1.6
Duodenal atresia	3	1.2
Microcephaly	3	1.2
Meatal stenosis	3	1.2
Craniorachischisis	3	1.2
Congenital inguinal hernia	3	1.2
Chest deformity	1	0.4
Ambiguous genitalia	1	0.4
Omphalocell	1	0.4
Total	253	100.0

## Discussion

Birth defects, also called CAs, occur at a rate of 1 in every 33 deliveries, although the degree of defect varies as some are minor, while others are major defects. In most cases, the major defects are the leading causes of high perinatal mortality and morbidity in developing and developed countries [24]. According to World Health Statistics, about 260,000 neonatal deaths are caused by BDs, accounting for 7% of all neonatal deaths globally [10]. However, it varies from 5% in south-east Asia to more than 25% in Europe [10]. It was also indicated that variation between-countries ranging from 4% in (Bangladesh, Equatorial Guinea, Ethiopia, Liberia, Mali, and Sierra Leone) to 8% in China [10]. In the present study, the overall proportion of BDs was 0.6%, which is lower than the result of a study (2%) in Addis Ababa [19]. The differences might be attributed to the study design, retrospective data from record reviews (in the present study) versus the primary data (in Addis Ababa), or might be related to socioeconomic differences between the two study populations within a different geographical location.

In this finding, the overall prevalence rate of BDs was 55 per 10,000 births, of which NTDs were the most prevalent constituting 73.5% of all the defects. A similar previous study also showed that the NTDs were the most prevalent types of BDs with a frequency of 32.5% in northwestern Ethiopia [25]. However, the proportions in our findings were higher than those of northwestern Ethiopia. The difference might be due to different study settings (six hospitals in this study) while only one referral hospital in the previous survey or discrepancy in the study population's socio-economic status.

Our findings showed that the prevalence rate of NTD was 40.05 per 10,000 births with predominant types of NTDs, namely: anencephaly (13.7 per 10,000 births), hydrocephalus (13.2 per 10,000 births), spina bifida (7.2 per 10,000 births), and meningomyelocele (3.9 per 10,000 births). The prevalence rate of NTD in the present study was twice higher (40.5 per 10,000 births) than the study done in China (20.1 per 10,000 births). On the other hand, the prevalence rate of spina bifida (7.2 per 10,000 births) was slightly less compared to the previous report (10.6 per 10,000 births) in China. This divergence might be because of racial, geographical, environmental, and genetic factors or due to multifactor inheritance attributes.

In this study, anencephaly and hydrocephalus were the most common types of BDs with a frequency of 25% and 24.6%, respectively. The remaining BDs identified in this study altogether make a prevalence rate of up to 14.3 per 10,000 births. In line with our findings, Abbey *et al*. [26] reported that the prevalence of major BDs at the University of Port Harcourt Teaching Hospital in the Niger Delta was 207 per 10,000 live births, where the central nervous system predominating at 27% of the total BDs. In both studies, the NTD was more prevalent than other BDs, revealing that NTD was the most prevalent throughout the world.

The study done in China identified that the prevalence rate of BD was 156 per 10,000 births [3], while another study in Korea showed the overall prevalence of a major BD was 446.3 per 10,000 births [27]. In both studies, the BDs seemed to be higher as compared to the present study. This difference might be due to differences in socio-demographic, registration and record-keeping, racial, or environmental factors.

Unlike the present study where the NTDs were found to be the most prevalent defects, a study done in China indicated that septal defects (138.2 per 10,000 births) were the most prevalent followed by congenital hip dislocation (652 per 10,000 births) [27]. The study done in Addis Ababa and Amhara region by Mola *et al*. [19] revealed that oro-facial defects (34.2%) were the most prevalent followed by NTDs (30.8%). In contrast, NTD (73.5%) was the most prevalent, followed by a gastrointestinal defect (13.4%) in the present study. This difference may occur due to socio-economic, lifestyle, or demographic differences between the two study populations or the level of exposure to the causative agents.

Although male - to - female ratio in the present study was nearly 1: 1 (51.8% and 48.2%), males seem to some extent more affected than females. In contrast, the survey done in northwest Nigeria indicated a female - to - male ratio of 1.4: 1 [28,29]. However, this needs further studies to justify the underlying conditions. According to the European surveillance of BDs (EUROCAT) report, the prevalence of major BDs from 2003-2007 was 239 per 10,000 births, of which 80% were delivered, 17.6% terminated by induced abortion, 2.5% died after birth, and 2% were stillbirths [27,29]. This report indicated that BDs were the significant causes of infant morbidity and mortality, and this being a considerable community burden, paying due attention is crucial.

**Strengths and limitations of this study:** we used high caseload hospitals where most cases of BDs are expected in southwestern Ethiopia. We evaluated the cases from a large population of which there were 45,951 deliveries during the study period. Hence, the study results can be generalized to the study population of the study area. One of the limitations of the present study is the nature of the study. i.e., it was a hospital-based retrospective record review where incomplete records or information missed part of the records from the document may lead to bias. The outcome may not represent the actual prevalence of BDs in the southwestern Ethiopian population. It is logical to anticipate that each hospital might not capture every piece of information. Hence, a further community-based study that may represent the prevalence of BDs for the entire community of the southwestern Ethiopian population needs to be conducted.

**Funding:** this study was financially supported by Addis Ababa University and Jimma University. The funding body did not play any role in designing the research, collection, analysis, and interpretation of the data, and write up of the manuscript.

## Conclusion

The present study has revealed that the prevalence rate of BD at birth was 55 cases per 10,000 births, which was high compared to other studies. Among twenty-one BDs identified in this study, the NTDs were the most prevalent. While all the rest constitute only a quarter of the total BDs identified in this study. The study also revealed that nearly equal proportions of BD occurred among males and females. The majority of the mothers who gave birth to BDs were younger than 35 years old. As BDs are the most prevalent in the study area, early planning, designing, and implementing prevention and management schemes are crucial. Particularly, there is a need for urgent prevention mechanism strategies to reduce the possible occurrences of the BDs which through family planning, youth education, antenatal care services, and rational and professional assisted medication use during pregnancy.

### What is known about this topic

- *The prevalence of BDs varies among different ethnic groups;*
- *Birth defects are one of the significant childhood health problems as well as the cause of infant mortality and morbidity;*
- *Only a few studies have been conducted in Ethiopia, and no study is conducted in southwestern Ethiopia*.

### What this study adds

- *The prevalence of birth defects among newborns in southwestern Ethiopia is 55 per 10,000 births;*
- *The most common types of birth defects are nueral tube defects;*
- *The status of the infant at birth, birth weight, and gestational age are found to be associated with types of birth defects*.

## References

[1] Butt F, Shahzad R, Pasha I (2013). Pattern and outcome of congenital anomalies and maternal risk factor association. Biomedica.

[2] Victor BP (2002). Preventing congenital anomalies in developing countries. Community Genet.

[3] Zhang X, Li S, Wu S, HaoX Guo S, Suzuki K, Yokomichi H, Yamagata Z (2012). Prevalence of birth defects and risk factor analysis from a population-based survey in inner Mongolia, China. BioMedical Centre Pediatrics.

[4] Ekwere OE, Rosie M, BobPaul A, Bamidele J, Olorunleke O, Sunday P (2011). A retrospective study of congenital anomalies presented at tertiary health facilities in Jos, Nigeria. International Journal of Pharmaceutical Science Invention.

[5] Ahmadzadeh A, Safikhani Z, AbdulahiM,Ahmadzadeh A (2008). Congenital malformations among live births at Arvand Hospital, Ahwaz, Iran. Pakistan Journal of Medical Sciences.

[6] WHO The sixty-third world assembly of the health. Geneva.

[7] Shawky RM, Sadik DI (2011). Congenital malformations prevalent among Egyptian children and associated risk factors. The Egyptian Journal of Medical Human Genetics.

[8] Delport SD, Christianson AL, Van Den Berg HJS, Wolmarans L, Gericke GS (1995). Congenital anomalies in black south African liveborn neonates at an urban academic hospital. South African Medical Journal.

[9] Parmar A, Rathod SP, Patel SV, Patel SM (2010). A study of congenital anomalies in newborns. National Journal of Integrated Research in Medicine.

[10] Rizk F, Salameh P, Hamadé A (2014). Congenital anomalies: Prevalence and risk factors. Universal Journal of Public Health.

[11] Mohamed AEK, Ehab AAB, Ibrahim L (2013). Pattern of congenital anomalies in newborn: A hospital-based study. Pediatric Reports.

[12] Taboo ZA (2012). Prevalence and risk factors for congenital anomalies in Mosul City. The Iraqi Postgraduate Medical Journal.

[13] Amany MA, Shadia A, Azza AA, Hassan MG (2011). Assessment of risk factors for fetal congenital anomalies among pregnant women at Cairo University Hospitals. Journal of American Science.

[14] Naeimeh T, Katayon Y, Nazila N (2010). The Prevalence of congenital malformations and its correlation with consanguineous marriages. Oman Medical Journal.

[15] Vinceti M, Malagoli C, Rodolfi R, Astolfi G, Rivieri F, Fiorini S (2006). Prevalence at birth of congenital anomalies in a population living around a modern municipal solid waste inciderator. Epidemiology.

[16] Ekwere OE, Rosie M, BobPaul A, Bamidele J, Olorunleke O, Sunday P (2011). A retrospective study of congenital anomalies presented at tertiary health facilities in Jos, Nigeria. International Journal of Pharmaceutical Science Invention.

[17] Waqas J, Farooq A, Taimoor J, Muhammad SM (2009). Prevalence of gross congenital malformations at birth in the neonates in a Tertiary Care Hospital. Epidemiology.

[18] Flores AL, Vellozzi C, Valencia D, Sniezek J (2014). Global burden of neural tube defects, risk factors, and prevention. Indian J Community Health.

[19] Taye M, Afewor M, Fantaye W, Diro E, Worku A (2018). Factors associated with congenital anomalies in Addis Ababa and the Amhara Region, Ethiopia: a case-control study. BMC Pediatrics.

[20] Golalipour MJ, Najafi L, Keshtkar AA (2010). Prevalence of anencephaly in Gorgan, northern Iran. Arch Iran Med.

[21] Neumann PE, Frankel WN, Letts VA, Coffin JM, Copp AJ, Bernfield M (1994). Multifactorial inheritance of neural tube defects: Localization of the major gene and recognition of modifiers in ct mutant mice. Nature Genetics.

[22] Dean AG, Sullivan KM, Soe MM (2013). OpenEpi: Open Source Epidemiologic Statistics for Public Health.

[23] Taye M, Afework M, Fantaye W, Diro E, Worku A (2019). Congenital anomalies prevalence in AddisAbaba and the Amhara region, Ethiopia: descriptive cross-sectional study. BMC Pediatrics.

[24] Rosano A, Botto LD, Botting B, Mastroiacovo P (2000). Infant mortality and congenital anomalies from 1950 to 1994: An international perspective. Journal of Epidemiology and Community Health.

[25] Adane F, Seyoum G (2018). Prevalence and associated factors of birth defects among newborns at referral hospitals in Northwest Ethiopia. Ethiopian Journal of Health Development.

[26] Abbey M, Oloyede OA, Bassey G, Kejeh B, Otaigbe BE, Opara PI (2017). Prevalence and pattern of birth defects in a tertiary health facility in the Niger Delta area of Nigeria. International Journal of Women Health.

[27] Jung-Keun K, Dirga KL, Hwan-Cheol K, Jong-Han L (2004). Trends in the prevalence of selected birth defects in Korea. International Journal of Environmenta Research and Public Health.

[28] Nnadi DC, Singh S (2017). The prevalence of neural tube defects in North-West Nigeria. Saudi Journal for Health Sciences.

[29] Singh S, Chukwunyere DN, Omembelede J, Onankpa B (2015). Foetal congenital anomalies: An experience from a tertiary health institution in north-west Nigeria (2011-2013). Niger Postgraduate Medical Journal.

